# Laser Printing of Plasmonic Nanosponges

**DOI:** 10.3390/nano10122427

**Published:** 2020-12-04

**Authors:** Sergey Syubaev, Stanislav Gurbatov, Evgeny Modin, Denver P. Linklater, Saulius Juodkazis, Evgeny L. Gurevich, Aleksandr Kuchmizhak

**Affiliations:** 1 Institute of Automation and Control Processes, Far Eastern Branch, Russian Academy of Sciences, 690041 Vladivostok, Russia; trilar@bk.ru (S.S.); gurbatov_slava@mail.ru (S.G.); 2 Far Eastern Federal University, 690041 Vladivostok, Russia; 3 CIC NanoGUNE BRTA, Avda Tolosa 76, 20018 Donostia-San Sebastian, Spain; e.modin@nanogune.eu; 4 Optical Sciences Center and ARC Training Centre in Surface Engineering for Advanced Materials (SEAM), School of Science, Swinburne University of Technology, John st., Hawthorn, VIC 3122, Australia; dlinklater@swin.edu.au (D.P.L.); saulius.juodkazis@gmail.com (S.J.); 5 School of Science, RMIT University, Melbourne, VIC 3000, Australia; 6 World Research Hub Initiative (WRHI), School of Materials and Chemical Technology, Tokyo Institute of Technology, 2-12-1, Ookayama, Meguro-ku, Tokyo 152-8550, Japan; 7 Laser Center (LFM), University of Applied Sciences Munster, Stegerwaldstraße 39, 48565 Steinfurt, Germany; gurevich@fh-muenster.de

**Keywords:** laser ablation, noble-metal films, magnetron sputtering, nanosecond laser pulses, porous nanostructures, plasmonics, nanosponges

## Abstract

Three-dimensional porous nanostructures made of noble metals represent novel class of nanomaterials promising for nonlinear nanooptics and sensors. Such nanostructures are typically fabricated using either reproducible yet time-consuming and costly multi-step lithography protocols or less reproducible chemical synthesis that involve liquid processing with toxic compounds. Here, we combined scalable nanosecond-laser ablation with advanced engineering of the chemical composition of thin substrate-supported Au films to produce nanobumps containing multiple nanopores inside. Most of the nanopores hidden beneath the nanobump surface can be further uncapped using gentle etching of the nanobumps by an Ar-ion beam to form functional 3D plasmonic nanosponges. The nanopores 10–150 nm in diameter were found to appear via laser-induced explosive evaporation/boiling and coalescence of the randomly arranged nucleation sites formed by nitrogen-rich areas of the Au films. Density of the nanopores can be controlled by the amount of the nitrogen in the Au films regulated in the process of their magnetron sputtering assisted with nitrogen-containing discharge gas.

## 1. Introduction

Three-dimensional (3D) percolated porous nanostructures made of noble metals and having large surface-to-volume ratio have drawn significant attention due to their remarkable physicochemical properties allowing to use them for various important applications ranging from the photo- or electro-catalysis, water splitting, hydrogen storage to bio- and chemosensing via surface-enhanced effects [1,2,3,4,5]. For most of the suggested applications, the surface-to-volume ratio defined by the distribution, density and size of the pores within the 3D nanostructure is of crucial importance.

Recently, optical properties of 3D porous nanostructures have become a hot topic [6,7,8,9,10]. Specifically, the porous Au nanoparticles (also referred to as nanosponges) were shown to demonstrate polarization-dependent scattering as well as to support long-lived electron emission associated with localized and propagating surface plasmon modes having remarkably high quality factors. These optical properties make such structures appealing for various optical and nonlinear optical applications including random lasing, enhanced photo-emission, harmonic and supercontinuum light generation, as well as single-molecule biosensing based on metal-enhanced fluorescence, surface-enhanced Raman scattering (SERS), infrared absorption (SEIRA), etc. Multiple sensing applications are benefited from the random (but highly dense) arrangement of the plasmon-mediated electromagnetic (EM) hot spots within the structure allowing to obtain the spectrally broadband signal enhancement over the entire visible and near-IR spectral range [11,12,13,14]. Indeed, incorporation of the nanopores into the bulk plasmonic nanostructures with a sub-wavelength overall size provides more intense SERS signal due to multiple hot spots and enlarged surface area increasing probability for analyte molecules to reach these hot spots [15,16,17,18]. Nevertheless, upon excitation with EM radiation, both the arrangement of the nanosized pores and the overall nanostructure geometry govern the resulting response [19,20]. From this point of view, the plasmonic properties of the resulting 3D porous nanostructures, their general geometric shape as well as porosity are to be adjusted simultaneously that still remains challenging.

State-of-the-art methods for porous nanostructure fabrication generally require complicated multi-step fabrication protocols as dealloying and soft- or hard-template synthesis [21,22,23,24,25,26,27], where accurate management of the reaction conditions (temperature, composition, precursors, etc.) is crucial. Furthermore, the minimization of the surface free energy typically leads to generally spherical-shaped nanostructures. Seed-mediated growth provides simple and versatile method allowing to produce arbitrary-shaped porous nanostructures [28]. However, it’s often problematically to obtain only one desired geometry because of internal structural variations of the seeds as well as local variations of the reaction environment. Alternatively, liquid-free lithography-based approaches as electron- or ion-beam milling [29] are suitable for high-precision formation of geometrically-diverse nanostructures. However, the need for upscaling of the fabrication procedure creates an economically justified barrier for lithography-based techniques being applied for porous nanostructure fabrication and large-area replication. In addition, post-processing is also required to impart porosity into the nanostructures.

Herein, we applied scalable easy-to-implement nanosecond (ns) laser ablation of nitrogen-rich Au films to fabricate parabola-shaped nanobumps containing multiple nanopores inside. The nanopores were found to originate from laser-induced explosive boiling and coalescence of the nucleation sites formed by nitrogen-rich areas of the Au film, while the nanopore density can be controlled by amount of nitrogen used as discharge gas for magnetron sputtering of the Au films. Most of the nanopores hidden beneath the nanobump surface can be further uncapped using gentle etching of the nanobumps by an Ar-ion beam to form 3D plasmonic nanosponges promising for various nonlinear optical and sensing applications.

## 2. Materials and Methods

### 2.1. Deposition of Au Films Assisted with Various Discharge Gases

Au films with a thickness of 150 ± 5 nm were deposited onto silica glass substrates without any adhesion sub-layer using a custom-built magnetron sputtering system and three discharge gases: argon, nitrogen and purified air. Deposition was performed at 10${}^{-2}$ mbar and fixed applied voltage of 2.5 kV. At the same time, the current was maintained at a constant value of 25 mA by dynamically adjusting the discharge gas pressure that allowed to fix the sputtering rate at ≈1 nm·s${}^{-1}$ for all gases.

### 2.2. Characterization of Au Films

The actual thickness and average roughness of the films were controlled by an atomic-force microscopy (AFM, Nano-DST, Pacific Nanotechnology, Santa Clara, CA, USA). Optical spectroscopic measurements performed with an integrating sphere spectrometer confirmed the identical reflectance for all Au films evaporated with different discharge gases (Cary 5000, Agilent Technologies, Santa Clara, CA, USA). The surface chemical composition of the Au films was carefully studied with X-ray Photoelectron Spectroscopy (XPS). XPS spectra were collected using a Kratos Axis Nova instrument (Kratos Analytical Inc., Manchester, UK) with a monochromatic Al Kα source (source energy 1486.69 eV) at a power of 150 W. Elemental identification was carried out using survey spectra collected at a pass energy of 160 eV with 1 eV steps. A Shirley algorithm was used to measure the background core-level spectra, and chemically distinct species in the high-resolution regions of the spectra were fitted with synthetic Gaussian Lorentzian components after removing the background (using the CasaXPS software, v. 2.3.15). High-resolution XPS scans were performed in the N1*s* and Au4*f* regions.

### 2.3. Fabrication of Porous Nanostructures and Nanosponges

Precise pointed ablation of the Au films was performed with second-harmonic (wavelength of 532 nm), ns (pulse duration of 7 ns) laser pulses generated by an Nd:YAG laser system (Brio GRM Gaussian, Quantel, France). The laser radiation was focused into a sub-micrometer spot on the sample surface using a dry objective (Nikon, 50x Plan Fluor, Tokyo, Japan) with a numerical aperture NA of 0.8 (optical spot diameter of 1.22λ(NA)${}^{-1}\approx$ 0.8 μm on the sample surface). The sample was mounted onto a PC-driven nanopositioning platform (ANT series, Aerotech Gmbh., Nurnberg, Germany) allowing spot-by-spot laser printing of the computer-generated patterns with the movement repeatability better than 100 nm. The laser fluence was monitored by a pyroelectric photodetector (Ophir Optronics, Jerusalem, Israel) and adjusted by a PC-driven attenuator (Standa, Vilnius, Lithuania). All nanostructures were produced under identical ambient conditions upon single-pulse laser irradiation.

Additionally, to reveal the nanopores hidden beneath the surface, the laser-printed nanobumps were also post-processed via etching with an accelerated Ar-ion beam (IM4000, Hitachi, Tokyo, Japan) at acceleration voltage of 3 kV, gas flow of 0.15 cm${}^{3}$/min and discharge current of 105 μA. Such parameters were previously calibrated to provide relatively slow removal rate of ≈1 nm/s [15,30], allowing to avoid excessive heating, melting or deformation of the Au film and laser-printed nanostructures.

### 2.4. Characterization of Laser-Printed Nanostructures

Scanning electron microscopy (SEM) was performed with a Helios Nanolab 450 FIB-SEM (Thermo Fisher Scientific, Waltham, MA, USA). High-resolution surface characterization was conducted at an accelerating voltage of 5 kV and electron beam current 100 pA. Signal channels with secondary (SE) and back-scattered electrons (BSE) were simultaneously collected and analysed. Despite the lack of topographical information, the escape depth for BSE is greater than that for SE improving material/density sensitivity. This allows visualisation of the nanopores under the surface with high contrast and spatial resolution.

To shed light onto the internal structure of the nanobumps (nanosponges), we involved a focused ion beam technique to prepare single cross-sectional cuts and serial cuts that were subsequently combined into a 3D reconstruction. After defining the area of interest, deposition (starting from electron beam-induced deposition to prevent surface damage and followed ion beam-induced deposition) of the Pt protective layers was performed. The thickness of the layers was chosen taking into account the morphology and smoothness of the certain laser-printed structure and varied between 150 nm (for nanobumps) and 1000 nm (for through holes). Slicing was carried out using an Ga-ion beam at an accelerating voltage of 30 kV and beam current of 30 pA. After producing subsequent FIB cut, high-resolution SEM image was automatically acquired at 5 kV and 50 pA. The resulting image had a field of view of 2.5×1.6μm at a 1536×1024 pixel resolution, which corresponds to the pixel size of 2.4×1.6 nm. A series of 111 slices were acquired and measured slice thickness was 12 nm with standard deviation of 6.54 nm. Further data processing (including alignment, filtration, and visualization) was performed using Aviso 8.1 software (Thermo Fisher Scientific, Waltham, MA, USA).

Three-dimensional finite-difference time-domain (FDTD) calculations were undertaken to reveal local structure of the EM fields excited near the isolated plasmonic nanosponge by linearly-polarized laser pump at 532, 632 and 1030 nm. The pump wavelengths within rather broad spectral range were chosen to highlight potential applications, where the enhanced and localized plasmon-mediated fields are highly demanding including SERS substrates and enhancement of nonlinear optical effects. Representative 3D model of the nanosponge was reconstructed using high resolution top- and side-view SEM images. The linearly polarized laser radiation was modeled to pump the nanosponge from the top. Elementary cell size was 1×1×1 nm${}^{3}$, while the computational volume was limited by the perfectly matched layers. Dielectric permittivity of Au was modeled according to the data from [31].

## 3. Results and Discussion

**Figure 1:**
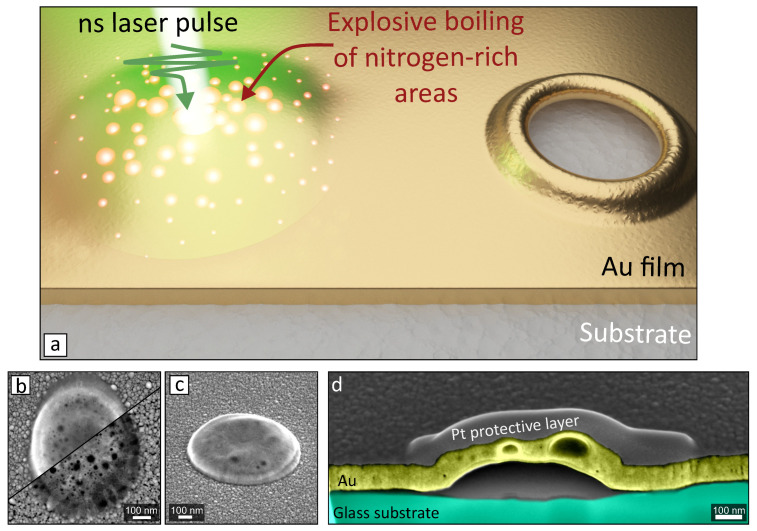
(**a**) Sketch illustrating direct laser printing of porous nanostructures on a glass-supported Au film. Single-pulse laser ablation at a near-threshold fluence produces a parabola-shaped nanobump (nanosponge), while the explosive boiling of randomly distributed nitrogen-rich sites create a nanoscale pores inside irradiated area. High-intense laser pulse drills a through hole where the nanopores can be found in the surrounding resolidified rim. (**b**,**c**) Representative top- and side-view SEM images of the isolated laser-printed nanosponge produced at F=0.15 J/cm${}^{2}$ in the Au film evaporated with nitrogen discharge gas. The top-view image is divided into two parts (recorded at different e-beam acceleration voltage) to illustrate multiple nanopores hidden beneath the nanobump surface. (**d**) False-color SEM images of the cross-sectional FIB cuts made through the center of such nanobump.

In this paper we considered three types of glass-supported Au films produced via magnetron sputtering in various discharges gases—argon, nitrogen and purified air (≈80% of nitrogen). Produced films had the same thickness (150±5 nm) and showed identical diffuse reluctance as well as AFM verified surface roughness of about 2±0.7 nm. This guarantied identical coupling of the incident ns-laser pulse energy to all types of metal films under study (Figure 1a). Such laser pulse induces thermalisation of charge carriers in the metal film that results in its local melting accompanied by detachment from the substrate via relaxation of the thermal-generated stress or evaporation at the interface between the film and the substrate [32,33]. At a pulse energy that is smaller than the ablation threshold (F${}_{th}\approx$ 0.17 J/cm${}^{2}$ [30,33,34,35]), detached metal shell resolidifies before its rupture forming parabola-shaped surface protrusion (also referred to as nanobump; Figure 1b,c). Typical laser-printed nanobump on the surface of 150-nm thick Au film sputtered with nitrogen discharge gas is illustrated by corresponding top- and side-view SEM images. The former image combines the signals from the SE and BSE detectors giving useful information regarding either a surface morphology or difference in chemical composition/density. The latter allowed to reveal nanoscale pores under the surface of the nanobump that can be also visualized by producing its FIB cross-sectional cuts (Figure 1d).

**Figure 2:**
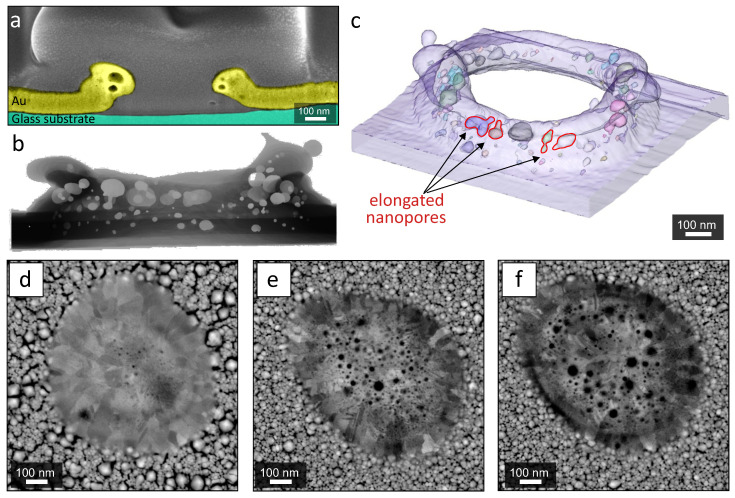
(**a**) False-color SEM images of cross-sectional FIB cuts made through the center of the microhole. The hole was produced at F=0.25 J/cm${}^{2}$ in the Au film evaporated with nitrogen discharge gas. (**b**,**c**) Distribution of the nanopores in the rim around a through hole visualized by tomographic 3D model reconstructed using serial FIB cuts. Several elongated irregular-shaped nanopores are highlighted in the figure. (**d**–**f**) Top-view SEM images of the nanobumps produced under single-pulse irradiation of the Au films (F≈0.15 J/cm${}^{2}$) produced with argon (**d**), purified air (**e**) and nitrogen (**f**) discharge gases, respectively.

At elevated laser fluence (F>0.23 J/cm${}^{2}$), rupture of the nanobump led to formation of the through hole in the metal film. In this case, the multiple nanopores can be identified within the resolidified rim surrounding the microhole as revealed by SEM visualization of the FIB cuts (see Figure 2a). Generally, for the fixed composition of the Au film, higher laser fluences produced the nanopores of the larger size. Similarly, the larger nanopores typically appeared closer to the nanobump center (see Figure 2d–e). Taking into account the Gaussian-shaped intensity profile of the irradiating laser beam, the nanopore formation appears to be driven by the local temperature (that will be discussed later in the text) that is higher in the metal film section coinciding with the beam center. To enrich information regarding the density and geometry of the nanopores, multiple FIB cuts were merged to build exact 3D model of through hole and the surrounding rim (Figure 2b,c). This 3D model clearly indicates broad size distribution of the nanopores ranging from 10 to 150 nm. Also, average size of the nanopores in the rim increases towards the center of the microhole. The larger nanopores can have irregular shape and reach the size ≈150 nm (Figure 2c), while the smaller nanopores far from the rim walls preserve spherical-like geometry.

In part, broad size distribution of the nanopores could be explained by merging (coalescence) of the closest nanopores growing from neighbouring randomly distributed nucleation centers. Origin of such nucleation centers will be discussed somewhat later in the paper in the context of chemical composition of the metal film fabricated with different discharge gases. Earlier coalescence was suggested as a leading mechanism of the water bubbles growing on dispersed gold nanoparticles heated by incident laser radiation [36,37]. As two bubbles with radii *r* merge, the gain in the surface energy $E_{+}=4\pi r^{2}\sigma \left(2-2^{2/3}\right)$ compensates the work of viscous forces needed to move the melt over a distance ∼r at a velocity, which can be estimated as v∼r/t. Here, *t* is the time needed for this melt relocation and σ=1.1N/m is the surface tension [38]. Using Newton’s law of viscosity to calculate the energy dissipation force $F=\eta vA/\delta_{h}$, where $A=4\pi r^{2}$—the surface area of the bubble, $\eta =4\times 10^{-3}\;Pa\;s$—viscosity of liquid gold [38], and $\delta_{h}∼r$—the characteristic length scale of the flow, we estimate the energy loss as $E_{-}=4\pi \eta r^{3}/t$. Equalizing $E_{+}$ and $E_{-}$ we estimate the time for two bubbles to merge t∼ηr/σ≈10–100ps (estimated for r=3–30 nm), which is less than the time gold film remains liquid.

This time can be estimated assuming that the heat accumulated by the illuminated spot is removed mostly by the thermal conductivity. The heated volume can be estimated as a cylinder of the radius r≈0.5μm (the typical lateral size of the nanobump) and the height h=0.15μm (the film thickness). The areas of the side $S_{side}=2\pi rh$ and the bottom $S_{bottom}=\pi r^{2}$ surfaces are comparable, but the thermal conductivity of gold $\lambda_{Au}=300$ W/(Km) is larger than that of glass $\lambda_{glass}=0.5$ W/(Km), so that only the heat flow through the side wall decreases the temperature of the molten surface. This heat flow can be estimated as $\frac{dE}{dt}=\lambda_{Au}S_{side}\nabla T\approx 2\pi h\lambda \left(T_{m}-T_{0}\right)$, where $T_{m}\approx 1.4\times 10^{3}$ K is the melting point of Au and $T_{0}=300$ K. This heat flow should remove the energy accumulated by the surface in one pulse $E_{p}=F\pi r^{2}$, hence the characteristic cooling time scale is $t∼\frac{Fr^{2}}{2h\lambda T_{m}}\approx 5\cdot 10^{-9}\;s$, i.e., is also comparable to the laser pulse duration. This estimation also agrees with previously reported studies [39]. Hence, coalescence of the bubbles provides a plausible explanation of the observed distribution of the pore sizes.

Noteworthy, different growth times of the pores can be also considered as alternative way to explain the large size variation of the nanopores. In particular, the pores started growing short after the onset of the melting had more time to develop than that ones started just before the resolidification. This assumption cannot be accepted because of the low growth velocity of the bubbles. Wang et al. [37] reported that during the initial bubble nucleation phase the bubble size *R* grows with time as $R\propto t^{1/6}$ (we notice that at later stages in degassed water this dependency drops to $R\propto t^{0.07}$, whereas in air-equilibrated water $R\propto t^{1/3}$). Hence, to get one order of magnitude difference in the nanopore radii, the times should differ by six orders magnitude. If the earliest possible nucleation starts after ten picoseconds after the start of the laser pulse (time comparable with the electron-phonon coupling times in metals), then the smaller pores formation must start several microseconds later, which is impossible because the resolidification happens on a time scale of several nanoseconds.

In a similar way, the broad size distribution can not be explained by local temperature fluctuations (pores at hot spots grow quicker) as the $R\left(t\right)\propto T^{1/3}$ [37]. Hence, three-orders-of-magnitude fluctuation of the local temperature in gold separated by a distances much less than one micrometer is obviously not possible. Spontaneous merging (coalescence) of several nanopores into the larger one can also stimulate rupture of the nanobumps and can explain formation of the through nanoholes previously reported for AuPd films processed with ns laser pulses [40]. The average pore size was observed to grow with the laser intensity, as it was shown in [15]. This can be explained by cooperative action of two mechanisms: (1) each pore grows more rapidly, since the temperature is higher and the pore radius $R\left(t\right)\propto T^{1/3}$. (2) The metal film remains molten for a longer time and the coalescence-based growth is stopped by the resolidification after a longer time interval t∝T, so that more pores can merge together. Assuming that the surface temperature is proportional to the intensity, we expect the average observed pore size to grow with the intensity.

Remarkably, the density and the average size of the nanopores started to grow from randomly distributed nucleation centers were found to depend on the type of discharge gas used for the film fabrication. More specifically, negligible amount of nanopores was found for the nanobumps produced on the surface of Au films sputtered with Ar (see Figure 2d). The maximal density of the nanopores was observed for the Au film evaporated with nitrogen discharge gas, decreasing for the Au film produced in purified air (Figure 2e,f). Taking into account same morphology and light absorbing characteristics of all mentioned Au films produced with different discharge gases, the amount of nitrogen in the film could be considered as a driving parameter that allows to control density of the nanopores. The molecular nitrogen can be bounded to the metal film surface as well as form chemically stable AuN phase upon magnetron sputtering as it was reported by several previous studies [41,42,43].

**Figure 3:**
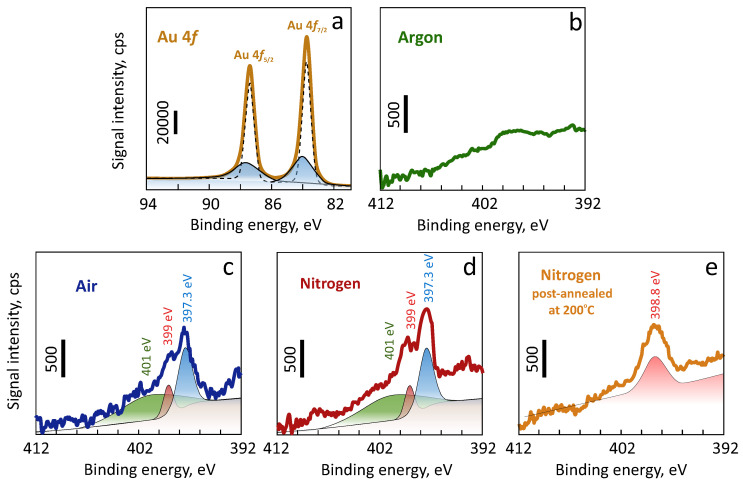
XPS characterization of the Au films produced using different discharge gases. (**a**) Representative Au4*f* photoemission spectra measured from the glass-supported Au film produced with nitrogen discharge gas. (**b**–**e**) N 1*s* core-level photoemission spectra of the Au films produced using magnetron sputtering assisted with various discharge gases: argon (**b**), purified air (**c**), nitrogen (**d**), nitrogen followed by thermal annealing at 200 ${}^{\circ }$C for 2 h (**e**). Deconvolution of the obtained spectral signal allowed for identification of several characteristic peaks highlighted by the colored areas in (**a**,**c**–**e**).

XPS measurements were performed to understand the effect of the discharge gas used for magnetron sputtering on the resulting chemical composition of the Au films (see Methods for details). Analyses of the deconvolved high resolution spectra taken in the N1*s* region revealed near-zero signal for films produced with Ar discharge gas. However, a pronounced signal for Au films fabricated with purified air and nitrogen (Figure 3b–d) were also observed. Deconvolution of the latter two spectra revealed several peaks with their binding energies at ≈401, 399 and 397.3 eV. The first high-energy peak can be assigned either to carbonitride [43] or to molecular nitrogen (that can be adsorbed on the metal film surface having multiple grains and cracks as well as be trapped beneath the surface [44,45]). The two remaining peaks can be attributed to chemically stable gold nitride phases AuN${}_{x}$ as well as to oxynitrides [41,42,43]. All of the different Au films demonstrated similar Au4*f* XPS spectra with low-intense shoulders (marked by blue areas in the Figure 3a) shifted towards larger binding energies near both low- (Au4$f_{7/2}$) and high-energy (Au4$f_{5/2}$) peaks. These shoulders can be indicative of stable chemical compounds like surface bonded carbon (AuC) or nitrogen (AuN${}_{x}$) as shown in previous studies [43]. However, the similarity of the signals obtain from different Au films does not clarify the exact chemical nature of these low-intense shoulders where signals from different compounds could overlap. To confirm such peak assignment, the Au film sputtered with nitrogen was further annealed at 200 ${}^{\circ }$C for 2 h. Thermal annealing was expected to remove thermodynamically unstable compounds (such as oxynitrides) as well as most of the molecular nitrogen from the near surface layer probed by XPS. N1*s* core-level spectra of the annealed Au film demonstrates the only remaining peak with a binding energy of 398.8 eV that can be presumably attributed to AuN${}_{x}$.

Considering the chemical composition of the Au film produced with different discharge gases we can suggest the following scenario regarding formation of the nanopores upon ns-pulse laser ablation. Such laser pulse with a near-threshold fluence rapidly heats up the Au film to temperatures above 10${}^{3}$ K. Such temperature jump is expected to remove all molecular nitrogen from the exposed area as well as induce an explosive boiling of the N- or AuN${}_{x}$-rich areas of the film. The data regarding melting/boiling temperatures of gold nitrides is weakly discussed in a literature. For example, annealing at 90 ${}^{\circ }$C was shown to remove AuN${}_{x}$ from the film [41], while present studies clearly showed signature of AuN${}_{x}$ remaining even after annealing at 200 ${}^{\circ }$C. Anyway, both melting/boiling temperatures of gold nitrides are expected to be much lower comparing to those for the pure gold. The light absorption should be initiated at the Au grain boundaries where also nitrogen adsorption should occur. The ionisation potential of Au is 9.23 eV while that of nitrogen is 14.53 eV (for O—13.62 eV). The laser pulse driven avalanche ionisation of gold is seeding the energy deposition which is evolving into run away ablation (melting, evaporation, ionisation).

**Figure 4:**
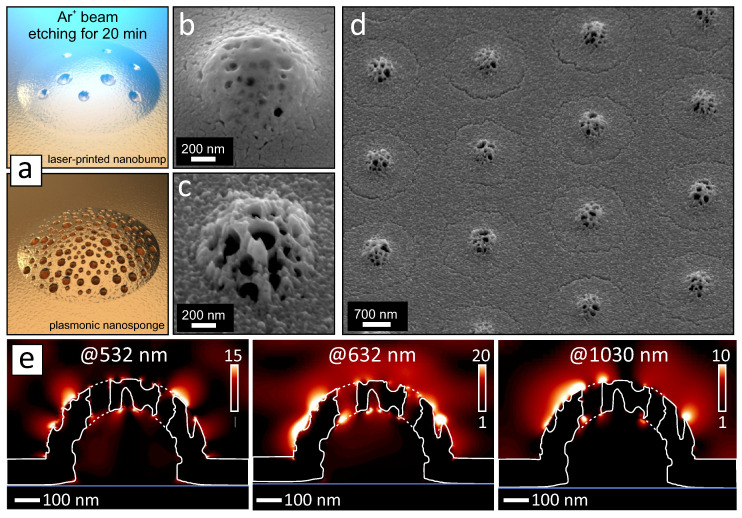
(**a**) Schematic illustration of the nanosponge fabrication. (**b**,**c**) Representative side-view SEM images comparing typical morphology of the nanobump before and after its etching with Ar-ion beam for 20 min. The nanobump was produced at F=0.165 J/cm${}^{2}$ on the surface of Au film evaporated with nitrogen discharge gas. (**d**) SEM image of the nanosponge array showing how the nanosponge morphology reproduces from pulse to pulse. (**e**) Calculated E${}^{2}$/E${}_{0}^{2}$ maps near the isolated Au nanosponge pumped at 532, 632 and 1030 nm.

The general geometry of the surface structure produced by direct laser ablation is defined by the laser irradiation parameters (fluence, pulse width and beam profile [46]) as well as by the thickness/composition of the metal film [33,47]. Our results clearly show that advanced chemical engineering of the metal film composition gives additional degree of freedom allowing to modify morphology of the laser-printed structures at the nanoscale, namely, incorporate the nanopores and control their density. However, a large amount of the nanopores is typically hidden beneath the top metal shell of the nanobump that limits the potential range of practically relevant applications. To make morphology of the laser-printed nanobumps more functional, we applied gentle etching of the laser-printed nanobumps with unfocused Ar-ion beam schematically illustrated on the Figure 4a. The processing parameters were calibrated to ensure gradual removal of the Au film without melting and deformation of the nanobumps geometry (see Materials and Methods). Two representative SEM images in the Figure 4b,c compare nanoscale surface morphology of the nanobump before and after its etching with Ar beam for 20 min. As can be seen, etching reveals the multiple hidden nanopores making the nanobump shell surface perforated with multiple through nanoholes. Besides the rather random arrangement of the nanopores (nanoholes), general geometry of the produced nanosponges reproduces well from pulse to pulse (see Figure 4d). From the simple geometrical consideration it is clear that the incident electromagnetic radiation can be efficiently absorbed by such nanosponges that produce enhanced electromagnetic fields via coupling to propagating and localized surface plasmons. Being combined with the mentioned broad size distribution of the nanopores, the 3D nanosponges with their lateral size of about visible-light wavelength are expected to be efficient for local EM field enhancement within rather broad spectral range spanning from visible to near-infrared. To illustrate this, we calculated squared electromagnetic field amplitude E${}^{2}$/E${}_{0}^{2}$ (E${}_{0}$ is the amplitude of the incident EM field) near the isolated plasmonic nanosponge pumped at 532, 632 and 1030 nm (see Materials and Methods). The cross-sectional E${}^{2}$/E${}_{0}^{2}$ maps shown in the Figure 4e indicate that the nanosponge supports densely arranged EM hot spots upon broadband EM excitation. Multi-fold enhancement of the EM field amplitude comes from coupling of the incident radiation to the localized plasmon resonances of the isolated nanoscale surface features on the top side of the Au shell (like nanopores, spikes and cracks clearly seen in the Figure 4c) as well as plasmons propagating within overall micro-scale sponge geometry. Propagating plasmons can couple to or re-excite the localized ones appearing as the localized EM hot spots on the opposite size of the opaque 150-nm thick Au shell. Owing to weak radiate decay, the certain localized plasmon modes in such plasmonic nanosponges were found to survive for a long time producing substantial field localization and enhancement [8]. Alternatively, most of the modes can dissipate via heating of the nanosponge. These features will potentially allow to use such nanosponges for various applications as thermo and nonlinear plasmonics (enhanced higher harmonic and white light generation), bio-imaging and visualization, photothermal conversion, sensing based on nonlinear optical effects as well as SEIRA- and SERS-based sensing [48,49,50,51,52].

## 4. Conclusions and Outlook

Here, we propose a simple approach allowing fabrication of porous plasmonic nanostructures using direct ns-laser ablation followed by Ar-ion beam etching. The nanopores were found to form through the explosive evaporation/boiling of the nitrogen-rich metal film areas exposed by a ns laser pulse. Detailed XPS and SEM analysis confirmed that the density of the nanopores correlates with the initial amount of nitrogen in the Au films that were fabricated by magnetron sputtering assisted with different discharge gases. Demonstrated unique porous nanostructures, parabola-shaped nanobumps perforated with multiple nanoholes, are expected to be useful for various applications where the plasmon-mediated EM fields are of mandatory importance as nonlinear plasmonic and chemo-/biosensing.

In a broader context, advanced chemical engineering of the metal film composition suggested in this paper being combined with an adjustment of the laser ablation process (as optimization of fluence, pulse width, laser beam shaping, etc.) is expected to provide facile way for fabrication of unique nanostructures. Also, elaborated strategy being applied to more complicated material combinations (like metal alloys or metal-dielectric materials) will further enrich the potential types of nanostructures as well as their application range [53,54,55]. For example, in a similar way nanosponges can be produced from co-sputtered noble-metal films forming nano-alloys with engineered permittivity as demonstrated for Au-Cu-Ag [56]. Along with plasmonic hot spot engineering, local potential defined by the nearest atomic composition was shown to modify physical and chemical adsorption of the analyte molecules affecting their characteristic SERS and SEIRA signals and sensor performance [57]. Insights into formation of metallic glasses and high entropy alloys with even large number of constituent materials [58] will benefit from a nanoscale control of re-melting and phase-explosions demonstrated in this work.
